# Self-organization of information processing in developing neuronal networks

**DOI:** 10.1186/1471-2202-16-S1-P221

**Published:** 2015-12-18

**Authors:** V Priesemann, J Lizier, M Wibral, ET Bullmore, O Paulsen, P Charlesworth, MS Schröter

**Affiliations:** 1 Department of Nonlinear Dynamics, Max Planck Institute for Dynamics and Self-Organization, Göttingen, Germany; 2 Bernstein Center for Computational Neuroscience, Göttingen, Germany; 3 School of Civil Engineering, University of Sydney, Sydney, Australia; 4 MEG Unit, Brain Imaging Center, Goethe University, Frankfurt am Main, Germany; 5 Behavioural & Clinical Neuroscience Institute, Department of Psychiatry, University of Cambridge, Cambridge CB2 3EB, UK; 6 Cambridgeshire and Peterborough NHS Foundation Trust, Cambridge CB21 5HH, UK; 7 GlaxoSmithKline, Immuno Psychiatry, Alternative Discovery and Development, Stevenage SG1 2NY, UK; 8 Department of Physiology, Development and Neuroscience, University of Cambridge, Physiological Laboratory, Downing Street, Cambridge CB2 3EG, UK

## 

Human brains possess sophisticated information processing capabilities, which rely on the coordinated interplay of billions of neurons. Despite recent advances in characterizing the collective neuronal dynamics, however, it remains a major challenge to understand the principles of how functional neuronal networks develop and maintain these processing capabilities. A popular hypothesis is that neuronal networks self-organize to a critical state [1-3], because in models, criticality maximizes information processing capacities [4-6]. This predicts that biological networks should develop towards a critical state during maturation, and at the same time processing capabilities should increase. We tested this hypothesis using multi-electrode spike recordings in mouse hippocampal and cortical neurons over the first four weeks *in vitro*. We showed that developing neuronal networks indeed increased their information processing capacities, as quantified by transfer entropy and active information storage [6-8]. The increase in processing capacity was tightly linked to decreasing the distance to criticality (correlation r = 0.68, p < 10^-9^; r = 0.55, p < 10^-6 ^for transfer and storage, respectively). Thereby our results for the first time demonstrate experimentally that approaching criticality with maturation goes in hand with increasing processing capabilities.
